# Recovery of Weak Factor Loadings When Adding the Mean Structure in Confirmatory Factor Analysis: A Simulation Study

**DOI:** 10.3389/fpsyg.2015.01943

**Published:** 2016-01-05

**Authors:** Carmen Ximénez

**Affiliations:** Department of Psychology, Autonomous University of MadridMadrid, Spain

**Keywords:** confirmatory factor analysis, recovery of weak factor loadings, mean structure, Monte Carlo simulation, psychometric models

## Abstract

This article extends previous research on the recovery of weak factor loadings in confirmatory factor analysis (CFA) by exploring the effects of adding the mean structure. This issue has not been examined in previous research. This study is based on the framework of Yung and Bentler (1999) and aims to examine the conditions that affect the recovery of weak factor loadings when the model includes the mean structure, compared to analyzing the covariance structure alone. A simulation study was conducted in which several constraints were defined for one-, two-, and three-factor models. Results show that adding the mean structure improves the recovery of weak factor loadings and reduces the asymptotic variances for the factor loadings, particularly for the models with a smaller number of factors and a small sample size. Therefore, under certain circumstances, modeling the means should be seriously considered for covariance models containing weak factor loadings.

Confirmatory factor analysis (CFA) is one of the most widely used statistical procedures in the social and behavioral sciences. In traditional CFA, researchers habitually analyze their models using only the covariance structures, thereby ignoring the associated mean structure. However, analyzing the associated mean structure can be relevant, as classical measurement models make assumptions involving latent means as well as covariance structures, and many CFA models with mean structure have been proposed (Sörbom, 1974; Millsap and Everson, 1991; Browne and Arminger, 1995; Little, 1997; Raykov, 2001). The advantages of analyzing the factor means compared to analyzing the means of the observed variables are well-documented in the literature (Kano et al., 1993; Yuan and Bentler, 1997, 2006; Yung and Bentler, 1999). Thus, the application of CFA models simultaneously analyzing the mean and covariance structure is widespread among researchers and practitioners (Ployhart and Oswald, 2004; Millsap and Meredith, 2007).

This article explores the advantages of simultaneously analyzing the mean and covariance structures, compared to analyzing a covariance structure alone, in the context of CFA models containing one or more weak factors. A *weak factor* is a factor that shows relatively little influence on the set of measured variables or is defined by small loading sizes (Briggs and MacCallum, 2003). A possible reason for finding such factors is the low reliability of the observed variables as a consequence of an inadequate wording of the items, which would result in a high measurement error and a small percentage of common variance. In such cases, these variables should be avoided. However, there are also situations in which estimating weak factors is important, and the unreliability problem is unavoidable because the items are well written. For instance, this may happen when measuring cognitive abilities or personality attributes that occupy a low position in the hierarchy of mental traits. One of the best known of such theories is Cattell's (1943) theory of intelligence, with Spearman's general factor (*g*) located at the top of the hierarchy and several major, minor, and specific group factors below *g*; and more recently, Ackerman's (1996) theory of the adult intellectual development process, personality, interests, and knowledge. In such cases, applied researchers must be aware of the consequences of working with factorial structures containing both strong and weak factors. Although past research has examined the conditions that affect the recovery of weak factors in the contexts of exploratory and CFA, no research has examined the advantages of simultaneously analyzing the mean and covariance structures, compared to analyzing a covariance structure alone for the recovery of weak factor loadings. This issue is important given the widespread use of these models in psychological research. Therefore, researchers performing a CFA with factorial structures that contain weak factors should be aware of the consequences of adding the mean structure to the estimation of the covariance model. Is the recovery of weak factors affected when adding the mean structure to the estimation of the CFA model? Do estimation methods equally recover the weak factors? Which conditions affect the weak factors' recovery and to what degree?

The present research shows that recovery of weak factor loadings is substantially improved when adding the associated mean structure to the estimation of the covariance model. A simulation study is presented in which recovery of weak factor loadings is studied under conditions of estimation method, sample size, constraints in the mean structure, and factor correlation. The study is based on the framework proposed by Yung and Bentler (1999), which proved that the reduction of asymptotic variance can be substantial for the estimation of factor loadings when the associated mean structure is added to the covariance structure model. The primary purpose of this simulation study was to examine the degree to which the recovery of weak factor loadings improves when adding the associated mean structure to the estimation of the CFA model in a range of conditions, compared to analyzing the covariance structure alone.

The article is organized as follows. First, theoretical aspects are reviewed, including the Yung and Bentler (1999) framework. Second, the design and results of a simulation study are presented. Finally, a general discussion summarizing the results and their practical implications for applied researchers is provided.

## Brief overview of recovery of weak factor loadings

Previous research has examined the conditions that affect the recovery of weak factors. Within the context of exploratory factor analysis (EFA), Briggs and MacCallum (2003) examined the performance of maximum likelihood (ML) and unweighted least squares (ULS) estimation methods to recover a known factor structure with relatively weak factors. They found that in situations with a moderate amount of error, ML often failed to recover the weak factor, whereas ULS succeeded. Within the context of CFA, the simulation studies by Ximénez (2006, 2009) explored the recovery of weak factor loadings under conditions of estimation method (ML vs. ULS), sample size (100, 300, and 500), loading size in the weak factor (0.25, 0.35, or 0.50), model specification (correct vs. incorrect by altering the number of factors), factor correlation (null and moderate), and model error (lack of fit between the population matrix and the model). The results showed that the recovery of weak factor loadings improved when factors were correlated and models were correctly specified. For models that were incorrectly specified, recovery was very poor when misspecification implied underfactoring, especially for models with orthogonal factors. In addition, the ULS method produced more convergent solutions and successfully recovered the weak factors in some instances in which ML failed.

Although previous research has examined a wide range of conditions that affect the recovery of weak factors, more research is needed to continue examining these effects under different study conditions. The topic of adding the mean structure to the CFA model has not been previously studied and deserves further research as it represents a realistic condition for researchers and practitioners. In this sense, the present study is aimed to examine the conditions that affect the recovery of weak factor loadings when the CFA model includes the mean structure and to provide guidance to practical researchers in the design of their studies.

## Background on adding the mean structure to the CFA model

The study is based on the framework proposed by Yung and Bentler (1999), which proved that adding the analysis of the associated mean structure in the CFA model improves the ML estimation of some covariance structure parameters because asymptotic variances for factor loadings are reduced. Given the relevance of their mathematical framework to this article, a brief summary of its demonstration is provided here.

The CFA model (Jöreskog and Sörbom, 1981) can be given as:

(1) x=Λξ+δ,

where ***x*** is a vector of *p* observed variables, **ξ** is a vector of *q* factors, **Λ** is a *p* x *q* matrix of factor loadings, and **δ** is a vector of *p* measurement error variables. For convenience, the CFA model traditionally assumes zero means for the observed and latent variables (i.e., E(**ξ**) = 0, E(**δ**) = 0, and E(***x***) = 0) and that E(**ξδ**) = 0. The covariance matrix for ***x***, denoted by **Σ**, is:

(2) $$\Sigma =\Lambda \Phi \Lambda \prime +\Theta_{\delta },$$

where **Φ** is the *q* x *q* covariance matrix of **ξ** and **Θ**_**δ**_ the *p* x *p* covariance matrix of **δ**.

The extended CFA model with mean structures was given by Sörbom (1974):

(3) $$x=\tau_{x}+\Lambda \xi +\delta .$$

The only difference between equations (1) and (3) is in the **τ**_***x***_ term, which is a *p* x 1 vector of constant intercept terms. As in the CFA model, the CFA model with mean structures (hereinafter referred to as CFA-MS model) assumes that E(**δ**) = 0 and E(**ξδ**) = 0, and the covariance matrix for ***x*** is given by Equation (2). However, it is not assumed that E(**ξ**) is zero. The mean of **ξ** is a parameter denoted by **κ**. Therefore, Equation (3) allows the comparison of the factor means. Under this formulation of the CFA model, by taking the expectation of Equation (3), the mean vectors of the observed variables is given by:

(4) $$E\left(x\right)=\mu_{x}=\tau_{x}+\Lambda \kappa .$$

Assuming the CFA-MS model defined in Equation (3), and that the mean and covariance structures of ***x*** under this model are those defined in Equations (4) and (2), respectively, let $\theta ={\left[vec(\Lambda )\prime ,v(\Phi )\prime ,diag(\Theta_{\delta })\prime \right]}^{\prime }$ be the vector of non-redundant parameters in **Λ**, **Φ**, and **Θ**_**δ**_, where *vec*(**Λ**), *v*(**Φ**), and *diag*(**Φ**_**δ**_) are vectorization operations for the corresponding matrices. **θ** is called the *vector of covariance structure parameters*. The vector for the parameters that are specific to the associated mean structure is: $\nu ={\left[\tau_{x}\prime ,\kappa \prime \right]}^{\prime }$. Here, for convenience, it is assumed that all the parameters in **θ** and **ν** are free and identified.

The question is how and why adding **ν** in the factor model may affect the estimation in **θ**. Assuming multivariate normality of the observed variables and the covariance structure model in Equation (2), the asymptotic covariance matrix of $\sqrt{n}(\hat{\theta }-\theta )$ is denoted as $V_{\theta \theta }=I_{c}^{-1}$, where $\hat{\theta }$ is the ML estimator of **θ**, and ***I**_c_* is the Fisher information matrix for **θ** (for the exact formulas of ***I**_c_*, see Jöreskog, 1974).

If the mean structure defined in Equation (4) is added in the analysis, then the information matrix for all parameters [**θ′**, **ν′**]′ can be written as:

(5) $$I_{mc}=\left[\begin{array}{cc} I_{\theta \theta } & I_{\theta v}\\ I_{v\theta } & I_{vv} \end{array}\right]=\left[\begin{array}{cc} I_{c}+I_{\theta \theta }^{*} & I_{\theta v}\\ I_{v\theta } & I_{vv} \end{array}\right],$$

where $I_{\theta \theta }^{*}$ is the added information about **θ** provided by the mean structure.

Yung and Bentler (1999) demonstrated that:

(6) $$I_{\theta \theta }^{*}=\left[\begin{array}{ccc} (\kappa \kappa^{\prime })⊗\Sigma^{-1} & 0 & 0\\ 0 & 0 & 0\\ 0 & 0 & 0 \end{array}\right],$$

(7) $$I_{\nu \theta }=\left[\begin{array}{ccc} \kappa^{\prime }⊗\Sigma^{-1} & 0 & 0\\ \kappa^{\prime }⊗(\Lambda^{\prime }\Sigma^{-1}) & 0 & 0 \end{array}\right],$$

(8) $$I_{\nu \nu }=\left[\begin{array}{cc} \Sigma^{-1} & \Sigma^{-1}\Lambda \\ \Lambda^{\prime }\Sigma^{-1} & \Lambda^{\prime }\Sigma^{-1}\Lambda \end{array}\right].$$

The arrangement of matrices (6) to (8) is associated with the order of the parameters as defined in **θ** and **ν**. The 0's inside the matrices (6) and (7) are null and conformable matrices. Examining Equation (6), it can be seen that there is added information in **Λ** provided by the mean structure, but there is not added information in **ν** and **Θ**_**δ**_. The submatrix (**κκ′**)⊗**Σ**^−1^, called the *added information about* **Λ** *provided by the mean structure*, contains information that may not be independent of the information regarding the parameters in **ν**, which are also added with the mean structure. If the estimation of **Λ** is improved when adding the mean structure, the estimation of **Φ** and **Θ**_**δ**_ may also be improved through their functional relations with **Λ** in the covariance structure in Equation (2). However, this only will happen if there is a better estimation of **Λ**.

Let $V_{\theta \theta }^{*}$ be the asymptotic covariance matrix of $\sqrt{n}(\hat{\theta }-\theta )$ under the CFA-MS model. Then, using the notation of Equation (5) and the inverse of the matrix, it is shown that:

(9) $$V_{\theta \theta }^{*}={\left[I_{c}+I_{\theta \theta }^{*}-I_{\theta v}{\left(I_{vv}\right)}^{-1}I_{v\theta }\right]}^{-1}.$$

**θ** can be estimated in two different situations: (1) under the covariance structure alone, and (2) adding the associated mean structure. If the inclusion of the associated mean structure leads to a smaller or an equal amount of asymptotic variance for $\hat{\theta }$, then it follows that:

(10) $$V_{\theta \theta }\geq V_{\theta \theta }^{*},$$

where ***V***_θθ_ and $V_{\theta \theta }^{*}$ are the asymptotic variances for the same parametric estimate under the covariance structure model alone and under the mean and covariance structure model, respectively. Proving (10) is equivalent to proving the following inequality:

(11) $$I_{c}\leq \left[I_{c}+I_{\theta \theta }^{*}-I_{\theta v}\;{\left(I_{vv}\right)}^{-1}I_{v\theta }\right].$$

After simplifying, equation (11) can be written as:

(12) $$\left[I_{\theta \theta }^{*}-I_{\theta v}\;{\left(I_{vv}\right)}^{-1}I_{v\theta }\right]\geq 0.$$

The left side of Equation (12) may be defined as *the net information about* **θ** *provided by the mean structure*. If the inequality established in Equation (12) is true (i.e., the net information about **θ** provided by the added mean structure is positive semi-definite), then there is a reduction of asymptotic variance for estimating any parametric functions of **θ** by adding the associated mean structure to the CFA model. Yung and Bentler (1999) stated that the necessary and sufficient condition for a non-null net gain of information about **θ** by adding the associated mean structure is that the mean structure is not saturated (i.e., the number of free parameters in **ν** is less than *p*) and **κ** is not a zero vector.

Yung and Bentler (1999) formulated this mathematical framework in their article and also provided two numerical examples to demonstrate that when the associated mean structure is added, the amount of reduction in the asymptotic variances is substantial. They examined the parameter recovery in a one-factor model with five observed variables in a single-group setup and in a two-group setup in the same model. They showed that adding the associated mean structure produces a notable reduction in the asymptotic variances for estimating the **Λ** parameters, and that this reduction is especially beneficial for factor loadings with smaller true values, the topic of interest of this article. Some authors have cited the framework by Yung and Bentler (1999), acknowledging the importance of adding the mean structure to the covariance model in ML estimation (e.g., Ogasawara, 2001, 2009; Liang and Bentler, 2004; Yuan et al., 2008). However, no research has examined the conditions of the design of the study that affect the estimation of the parameters when adding the mean structure to the covariance model. The present study addresses these issues in the context of CFA models containing weak factor loadings and provides specific recommendations concerning the benefits of adding the mean structure in such models.

## Monte carlo simulation study

The guidelines for Monte Carlo simulation designs in structural equation models recommended by Skrondal (2000) and Boomsma (2013) are used to present the design of the simulation study.

### Step 1: Research hypothesis and theoretical framework

This study explores the effects of the estimation method, sample size, constraints in the model, and factor correlation on the recovery of weak factor loadings, and on the relative reduction of asymptotic variances for the factor loadings obtained by adding the mean structure. The study is based on the framework proposed by Yung and Bentler (1999) and examines the hypothesis that the recovery of weak factor loadings improves when the associated mean structure is added to the covariance structure model, and that the reduction in the asymptotic variance is substantial for the estimation of weak factor loadings. Given that this issue has not been examined in previous research, one of the aims of the study is to examine the degree to which the recovery of weak factor loadings improves when adding the associated mean structure to the estimation of the CFA model in a range of conditions.

### Step 2: Experimental design

#### Population models

Following Boomsma's (2013) recommendations, the choice of the population models is based on previous Monte Carlo research on the recovery of weak factor loadings to increase the comparability of the experimental results and contribute to their external validity. The generating models are defined on the basis of the models used in Ximénez (2006), which include 12 measured normal variables and models with one, two, and three factors, of which one of the factors is relatively weak. A one-factor model has been included to examine the recovery of a single weak factor when adding the mean structure, and to compare the results to those of the examples provided by Yung and Bentler (1999), which referred only to one-factor models. However, as models with two or three factors would be encountered more often in practice, a study of how the weak factor loadings are recovered in the presence of stronger factors when adding the mean structure to the CFA model is also included. The theoretical values of the parameters for each factorial structure are summarized in the upper section of Table 1.

**Table 1:** **θ and ν true parameters of generating models**.

	**One-factor model**				**Two-factor model**						**Three-factor model**					
	**Factor 1**				**Factor 1**			**Factor 2**			**Factor 1**		**Factor 2**		**Factor 3**	
**COVARIANCE STRUCTURE:**																
λ_1_*_*j*_*	0.30				0.80			0			0.95		0		0	
λ_2_*_*j*_*	0.30				0.80			0			0.95		0		0	
λ_3_*_*j*_*	0.30				0.80			0			0.95		0		0	
λ_4_*_*j*_*	0.30				0.80			0			0.95		0		0	
λ_5_*_*j*_*	0.30				0.80			0			0.95		0		0	
λ_6_*_*j*_*	0.30				0.80			0			0		0.70		0	
λ_7_*_*j*_*	0.30				0.80			0			0		0.70		0	
λ_8_*_*j*_*	0.30				0			0.30			0		0.70		0	
λ_9_*_*j*_*	0.30				0			0.30			0		0.70		0	
λ_10_*_*j*_*	0.30				0			0.30			0		0		0.30	
λ_11_*_*j*_*	0.30				0			0.30			0		0		0.30	
λ_12_*_*j*_*	0.30				0			0.30			0		0		0.30	
ϕ_12_					0 or 0.50						0 or 0.50					
ϕ_13_											0 or 0.50					
ϕ_23_											0 or 0.50					
**MEAN STRUCTURE:**																
	***T***	**C1**	**C2**	**C3**	***T***	**C1**	**C2**		**C3**	**C4**	***T***	**C1**	**C2**	**C3**	**C4**	**C5**
τ_1_	3	0	0	0	8	0	0		0	0	9.5	0	0	0	0	0
τ_2_	3	^*^	0	^*^	8	^*^	0		0	^*^	9.5	^*^	0	0	^*^	^*^
τ_3_	3	^*^	0	0	8	^*^	0		0	^*^	9.5	^*^	0	0	^*^	^*^
τ_4_	3	^*^	0	^*^	8	^*^	0		0	^*^	9.5	^*^	0	0	^*^	^*^
τ_5_	3	^*^	0	0	8	^*^	0		0	^*^	9.5	^*^	0	0	^*^	^*^
τ_6_	3	^*^	0	^*^	8	^*^	0		0	^*^	7	0	0	0	0	0
τ_7_	3	^*^	0	0	8	^*^	0		0	^*^	7	^*^	0	^*^	0	^*^
τ_8_	3	^*^	0	^*^	3	0	0		0	0	7	^*^	0	^*^	0	^*^
τ_9_	3	^*^	0	0	3	^*^	0		^*^	0	7	^*^	0	^*^	0	^*^
τ_10_	3	^*^	0	^*^	3	^*^	0		^*^	0	3	0	0	0	0	0
τ_11_	3	^*^	0	0	3	^*^	0		^*^	0	3	^*^	0	^*^	^*^	0
τ_12_	3	^*^	0	^*^	3	^*^	0		^*^	0	3	^*^	0	^*^	^*^	0
κ_1_	6	^*^	^*^	^*^	12	^*^	^*^		^*^	^*^	12	^*^	^*^	^*^	^*^	^*^
κ_2_					6	^*^	^*^		^*^	^*^	8	^*^	^*^	^*^	^*^	^*^
κ_3_											6	^*^	^*^	^*^	^*^	^*^

*T refers to the true value of the generating models for **τ**_**x**_ and **κ**; and C1, C2, C3, C4, and C5 are the corresponding constraints for each model*.

* *indicates that the parameter is free*.

#### Experimental factors and response variables

The independent variables are estimation method (*M*), sample size (*N*), constraints in the model (*C*), and factor correlation (*CO*). Two estimation methods were considered: maximum likelihood (ML) and unweighted least squares (ULS). Although Yung and Bentler (1999) only considered ML estimation, this study includes ULS estimation because previous research has demonstrated that ML sometimes fails to recover the weak factor loadings when ULS succeeds; the interest in this study lies in exploring if this effect holds when adding the mean structure to the CFA model. Following Boomsma (1982), the smallest sample size chosen was *N* = 100. Sample sizes of 300 and 500 were used to approximate medium and relatively large sample sizes. Two levels of factor correlation were chosen (null: 0 and moderate: 0.50). Previous research has demonstrated that the recovery of weak factor loadings improves when factors are correlated. Thus, another topic of interest is to examine if this effect holds when adding the mean structure to the CFA model. Finally, several constraints were defined for the mean structure of each corresponding model. The theoretical values of the parameters for the mean structure are summarized in the lower section of Table 1. The one-factor model reflects the case in which the researcher is interested in checking the unidimensionality of a test, a classical measurement assumption in many psychometric models.

Following Millsap and Everson (1991), constraints on the latent mean structure were imposed to reflect measurement model assumptions. Three constraints were defined. C1 refers to the saturated model, where λ_11_ is fixed to unity and its associated intercept τ_1_ is fixed to zero for identification purposes, and the common factor mean and the remaining intercepts are estimated. C2 represents the *tau-equivalent model*, where the factor mean is estimated and the **τ**_***x***_ is a null vector. This also reflects the situation in which all the items in the test have the same units of measurement (e.g., a five-point Likert scale) and the test is unidimensional. Finally, C3 is somewhat similar to the *essentially congeneric model* of Millsap and Everson (1991), where the factor mean is non-zero and some elements of the **τ**_***x***_ vector are null and the other ones are estimated. Besides the study of recovery of weak factor loadings in classical measurement models, and following previous research on the recovery of weak factors, two- and three-factor models were also considered. Similar constraints to those in the one-factor model were defined. In the two-factor model, C1 refers to the saturated model, C2 to the model in which the **τ**_***x***_ is a null vector and it is assumed that all items have the same units of measurement, C3 is the model in which the **τ**_***x***_ elements are null in the strong factor, and C4 is the model in which the null **τ**_***x***_ elements are those of the weak factor, assuming that the items in the weak factor have the same units of measurement. Similarly, in the three-factor model, C1 is the saturated model, C2 the model in which the **τ**_***x***_ is a null vector, C3 and C4 the models in which the null **τ**_***x***_ elements are those of the first and second strong factors, respectively, and C5 is the model in which the null **τ**_***x***_ elements are those of the weak factor, assuming that the items in the weak factor have the same units of measurement.

Finally, the dependent variables are the recovery of weak factor loadings and the relative reduction of asymptotic variances for the factor loadings obtained by adding the mean structure.

### Step 3: Executing the simulations

The population factor structures defined in the upper section of Table 1 were used as the basis to simulate the sample covariance matrices. One thousand sample covariance matrices were simulated with the PRELIS 2 program of Jöreskog and Sörbom (1996a) for each model.

### Step 4: Estimation and replication

A CFA was conducted on each simulated sample covariance matrix using ML and ULS estimation. The parameter estimates were computed with the LISREL 8.80 program of Jöreskog and Sörbom (1996b). The sample factor solutions were evaluated to determine how the recovery of weak factor loadings and the asymptotic variances for the factor loadings are affected by the independent variables of the study. Simulation and estimation were repeated for 156,000 replications.

### Step 5: Analyses of output

Non-convergent solutions were deleted to study the effects of the independent variables on the recovery of the weak factor loadings. The operational definition employed was that of the LISREL program: failure to reach convergence after 250 iterations (see Jöreskog, 1967, p. 460). Moreover, Heywood cases were detected and deleted in each of the cells of the design.

The recovery of the weak factor loadings was assessed by examination of the correspondence between the theoretical loading and the estimated one. A measure of correspondence, the root mean square deviation (*RMSD*; Levine, 1977) was calculated for each factor in the theoretical model:

(13) $$RMSD_{k}=\sqrt{\sum_{i\;=\;1}^{p}{\left(\lambda_{ik(t)}-\lambda_{ik(e)}\right)}^{2}/p},$$

where *p* is the number of variables that define the factor *k*, λ_*ik*(*t*)_ is the theoretic loading for the observed variable *i* of the factor *k*, and λ_*ik*__(__*e*__)_ the corresponding loading obtained from the simulation data. *RMSD* reaches a minimum of zero for a perfect pattern-magnitude match and a maximum of two, when all loadings are equal to unity but of opposite signs. Intermediate values are difficult to interpret. In practice, it is considered that *RMSD* values below 0.20 are indicative of a satisfactory recovery.

Both descriptive and inferential statistical procedures were used for the evaluation of the research findings. Following Skrondal (2000), a simple metamodel is used to analyze the results, which includes only the main and the double interaction effects of each independent variable on the dependent variable.

For the two- and three-factor models, the following model was tested:

(14) $$\begin{array}{l} RMSD=\mu +M+N+C+CO+M^{*}N+M^{*}C+M^{*}CO+\\ \;+N^{*}C+N^{*}CO+C^{*}CO, \end{array}$$

where: *RMSD*: root mean square deviation

*M*: method (ML vs. ULS)
*N*: sample size (100, 300, and 500)
*C*: constraints imposed in each model (4, 5 or 6, depending on the model)
*CO*: correlation between factors (0 or 0.50)

For the one-factor model the metamodel includes all terms of Equation (14) except those that refer to *CO*. A separate ANOVA was conducted to test the effects included in the metamodel for the one-, two-, and three-factor models. All effects are viewed as independent. As the large sample size (156,000 replications) can cause even negligible effects to be statistically significant, the explained variance associated with each of the effects was also calculated, measured by the η^2^ statistic. The interpretation guidelines suggested by Cohen (1988) were adopted: values of η^2^ from 0.05 to 0.09 indicate a small effect; from 0.10 to 0.20 a medium effect; and above 0.20 a large effect. Multiple comparisons were also conducted for the effects that were shown to be statistically and practically significant.

The relative reduction of asymptotic variances for the factor loadings obtained by adding the mean structure of the model was measured by the *ARE* index of Yung and Bentler (1999):

(15) $$ARE=\frac{Var{\left(\sqrt{n}\hat{\Lambda }\right)}_{CFA-MS}}{Var{\left(\sqrt{n}\hat{\Lambda }\right)}_{CFA}},$$

where *Var* is the corresponding asymptotic variance for $\sqrt{n}\hat{\Lambda }$ in each model. Yung and Bentler (1999) suggested that if the mean structures lead to a reduction of asymptotic variance for a particular factor loading estimate, the corresponding *ARE* measure should be less than 1, indicating that the estimation using the covariance structure alone is not efficient, compared to the model with mean and covariance structures. The same metamodel as in Equation (14) was used to test the effects of the independent variables on the *ARE* index by an ANOVA.

## Results

### Non-convergence and heywood cases

Of the 156,000 solutions, 14,324 (9.2%) were non-convergent, and 10,437 (6.7%) presented Heywood cases. The proportion of convergent and proper solutions was higher for the one-factor model, which obtained 98% proper solutions, whereas the two- and three-factor models obtained 94 and 88%, respectively. The proportion of convergent and proper solutions was higher when the factors are correlated, the sample size is increased, and the model includes the mean structure. In addition, there were more convergent and proper solutions with the ULS estimation method. On the one hand, these results are congruent with previous research, and on the other, they show that adding the mean structure to the CFA model favors the occurrence of convergent and proper solutions.

### Recovery of weak factor loadings

The left-hand side of Table 2 shows the summary statistics for *RMSD* under the study conditions and Table 3 presents the corresponding results of the ANOVA.

**Table 2:** **Descriptive statistics for the *RMSD* and *ARE* Measures**.

	***RMSD***												***ARE***											
	**ML**						**ULS**						**ML**						**ULS**					
	***N*** = 100		***N*** = 300		***N*** = 500		***N*** = 100		***N*** = 300		***N*** = 500		***N*** = 100		***N*** = 300		***N*** = 500		***N*** = 100		***N*** = 300		***N*** = 500	
**Model**	***M***	***S.D***	***M***	***S.D***	***M***	***S.D***	***M***	***S.D***	***M***	***S.D***	***M***	***S.D***	***M***	***S.D***	***M***	***S.D***	***M***	***S.D***	***M***	***S.D***	***M***	***S.D***	***M***	***S.D***
**1F**																								
CFA	0.30	0.11	0.23	0.08	0.21	0.06	0.29	0.11	0.23	0.08	0.21	0.07												
CFA-MS-C1	0.15	0.06	0.08	0.02	0.06	0.01	0.14	0.06	0.08	0.02	0.06	0.01	0.43	0.07	0.34	0.10	0.35	0.11	0.43	0.05	0.35	0.12	0.33	0.12
CFA-MS-C2	0.04	0.06	0.02	0.01	0.01	0.01	0.03	0.05	0.02	0.01	0.01	0.01	0.08	0.01	0.05	0.02	0.00	0.00	0.06	0.02	0.03	0.03	0.00	0.00
CFA-MS-C3	0.11	0.04	0.06	0.02	0.04	0.01	0.10	0.03	0.06	0.02	0.04	0.01	0.23	0.15	0.19	0.15	0.22	0.15	0.20	0.15	0.19	0.16	0.16	0.19
**2F (*CO* = 0)**																								
CFA	0.20	0.12	0.11	0.06	0.08	0.03	0.19	0.11	0.11	0.05	0.08	0.03												
CFA-MS-C1	0.20	0.12	0.11	0.04	0.08	0.03	0.19	0.11	0.11	0.04	0.08	0.03	1.00	0.00	0.91	0.11	1.00	0.00	0.65	0.16	0.91	0.04	0.98	0.06
CFA-MS-C2	0.05	0.05	0.01	0.01	0.02	0.02	0.04	0.06	0.01	0.01	0.02	0.02	0.10	0.01	0.13	0.02	0.13	0.00	0.16	0.05	0.13	0.00	0.13	0.01
CFA-MS-C3	0.20	0.10	0.01	0.01	0.08	0.03	0.19	0.10	0.01	0.01	0.08	0.03	0.94	0.13	0.91	0.11	0.98	0.06	0.60	0.11	0.89	0.04	0.98	0.06
CFA-MS-C4	0.05	0.05	0.03	0.03	0.02	0.02	0.04	0.06	0.03	0.02	0.02	0.02	0.10	0.01	0.13	0.02	0.13	0.00	0.18	0.05	0.07	0.00	0.13	0.01
**2F (*CO* = 0.50)**																								
CFA	0.18	0.12	0.09	0.03	0.07	0.02	0.17	0.09	0.09	0.03	0.07	0.02												
CFA-MS-C1	0.17	0.10	0.09	0.03	0.07	0.02	0.16	0.08	0.09	0.03	0.07	0.02	1.00	0.01	0.96	0.06	1.00	0.00	0.89	0.02	1.00	0.00	1.00	0.00
CFA-MS-C2	0.08	0.11	0.04	0.03	0.04	0.02	0.07	0.08	0.04	0.03	0.04	0.02	0.45	0.04	0.24	0.02	0.20	0.00	0.29	0.04	0.10	0.01	0.17	0.02
CFA-MS-C3	0.17	0.10	0.09	0.03	0.07	0.02	0.16	0.08	0.09	0.03	0.07	0.02	0.98	0.02	0.96	0.06	1.00	0.00	0.88	0.03	1.00	0.00	1.00	0.00
CFA-MS-C4	0.08	0.10	0.04	0.03	0.04	0.02	0.07	0.08	0.04	0.03	0.04	0.02	0.43	0.04	0.24	0.02	0.20	0.00	0.31	0.05	0.10	0.01	0.17	0.02
**3F (*CO* = 0)**																								
CFA	0.29	0.23	0.19	0.26	0.14	0.12	0.29	0.23	0.19	0.16	0.14	0.12												
CFA-MS-C1	0.25	0.17	0.16	0.12	0.12	0.09	0.25	0.19	0.16	0.12	0.12	0.09	0.97	0.01	0.43	0.12	0.66	0.31	0.59	0.24	0.65	0.25	0.69	0.32
CFA-MS-C2	0.08	0.06	0.05	0.04	0.04	0.03	0.08	0.06	0.05	0.06	0.04	0.04	0.11	0.02	0.05	0.03	0.06	0.01	0.13	0.02	0.10	0.01	0.09	0.02
CFA-MS-C3	0.25	0.18	0.16	0.13	0.12	0.09	0.25	0.17	0.16	0.12	0.12	0.09	0.98	0.02	0.43	0.09	0.66	0.31	0.54	0.23	0.63	0.27	0.69	0.34
CFA-MS-C4	0.25	0.26	0.16	0.18	0.12	0.09	0.25	0.18	0.16	0.13	0.12	0.09	0.99	0.04	0.55	0.04	0.66	0.28	0.55	0.23	0.64	0.27	0.71	0.35
CFA-MS-C5	0.08	0.07	0.05	0.04	0.04	0.03	0.08	0.06	0.05	0.06	0.04	0.03	0.12	0.02	0.05	0.03	0.06	0.01	0.11	0.02	0.100	0.01	0.09	0.02
**3F (*CO* = 0.50)**																								
CFA	0.17	0.08	0.11	0.04	0.10	0.03	0.15	0.07	0.11	0.04	0.10	0.03												
CFA-MS-C1	0.16	0.08	0.11	0.04	0.10	0.03	0.15	0.07	0.11	0.04	0.10	0.03	1.00	0.000	1.00	0.00	1.00	0.00	0.97	0.03	1.00	0.00	1.00	0.00
CFA-MS-C2	0.11	0.08	0.09	0.04	0.09	0.04	0.11	0.07	0.09	0.04	0.09	0.03	0.49	0.07	0.33	0.00	0.25	0.00	0.38	0.02	0.33	0.00	0.25	0.00
CFA-MS-C3	0.16	0.08	0.11	0.04	0.10	0.03	0.15	0.06	0.11	0.04	0.10	0.03	1.00	0.00	1.00	0.00	1.0	0.00	0.92	0.02	1.00	0.00	1.00	0.00
CFA-MS-C4	0.16	0.08	0.11	0.04	0.10	0.03	0.15	0.07	0.11	0.04	0.10	0.03	0.95	0.02	1.00	0.00	1.0	0.00	0.92	0.03	1.00	0.00	1.00	0.00
CFA-MS-C5	0.11	0.08	0.09	0.04	0.09	0.04	0.11	0.07	0.09	0.04	0.09	0.04	0.46	0.06	0.33	0.00	0.25	0.00	0.42	0.02	0.33	0.00	1.00	0.00

**Table 3:** **ANOVA Results for the *RMSD* measure in the Monte Carlo study**.

	**One-factor model**				**Two-factor model**				**Three-factor model**			
	***df***	***F***	***Prob***.	**η^2^**	***df***	***F***	***Prob***.	**η^2^**	***df***	***F***	***Prob***.	**η^2^**
*M*	1	11.304	< 0.001	0.001	1	18.053	< 0.001	0.000	1	2.319	0.128	0.000
*N*	2	3115.575	< 0.001	0.210	2	7948.093	< 0.001	0.221	2	338.005	< 0.001	0.111
*C*	3	20726.20	< 0.001	0.726	4	4831.532	< 0.001	0.257	5	161.652	< 0.001	0.113
*CO*	–	–	–	–	1	155.743	< 0.001	0.003	1	139.136	< 0.001	0.020
*M* ^*^ *N*	2	7.630	< 0.001	0.001	2	23.342	< 0.001	0.001	2	1.626	0.197	0.000
*M* ^*^ *C*	3	0.178	0.911	0.000	4	0.145	0.965	0.000	5	1.244	0.286	0.000
*M* ^*^ *CO*	–	–	–	–	1	3.341	0.068	0.000	1	0.000	0.982	0.000
*N* ^*^ *C*	6	168.549	< 0.001	0.041	8	361.594	< 0.001	0.149	10	11.452	< 0.001	0.112
*N* ^*^ *CO*	–	–	–	–	2	63.187	< 0.001	0.002	2	66.483	< 0.001	0.002
*C* ^*^ *CO*	–	–	–	–	4	239.886	< 0.001	0.117	5	72.894	< 0.001	0.116
Error	23472	(0.003)			55874	(0.004)			62247	(0.105)		
Total	23490			0.747	55904			0.510	62282			0.430

Where M is method (ML vs. ULS), N is sample size (100, 300, or 500), C is type of constraint (see Table 1), and CO is correlation between factors (0 or 0.50). Values in parentheses represent mean squared errors.

**Figure 1:**
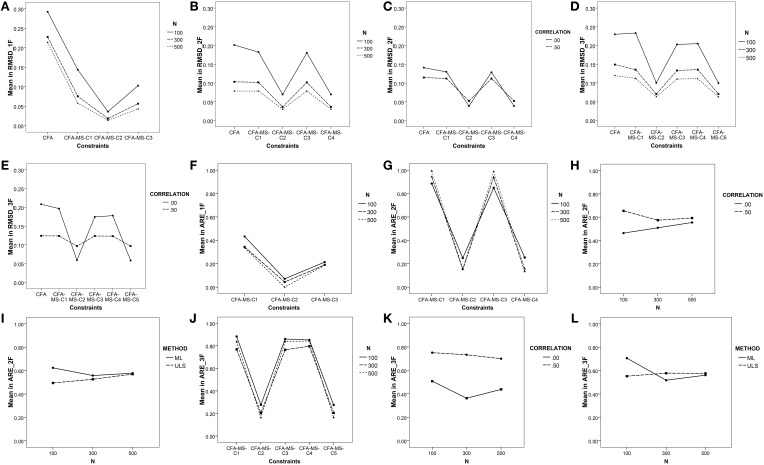
**Graphical representation of the strongest double interaction effects found on the recovery of weak factor loadings and the *ARE* measure**. **(A)** NxC interaction and RMSD (1F); **(B)** NxC interaction and RMSD (2F); **(C)** CxCO interaction and RMSD (2F); **(D)** NxC interaction and RMSD (3F); **(E)** CxCO interaction and RMSD (3F); **(F)** NxC interaction and ARE (1F); **(G)** NxC interaction and ARE (2F); **(H)** NxCO interaction and ARE (2F); **(I)** MxN interaction and ARE (2F); **(J)** NxC interaction and ARE (3F); **(K)** NxCO interaction and ARE (3F); **(L)** MxN interaction and ARE (3F).

As shown in Table 3, in the one-factor model all main effects and nearly all double interaction effects are statistically significant. The largest effects found are due to the *constraints imposed in the model* (η^2^ = 0.73) and *sample size* (η^2^ = 0.21) main effects. The *N*x*C* interaction also produced a small effect (η^2^ = 0.04). Figure 1A illustrates the *N*x*C* interaction. As can be seen, the average values of *RMSD* for the CFA model are indicative of a poor recovery in all sample sizes, especially in the smallest (*N* = 100). However, recovery is satisfactory when the associated mean structure is added to the covariance model. The recovery of weak factor loadings is satisfactory in all the constraints defined for the mean structure of the unidimensional model. The best results are for the C2 constraint, which represents the *tau-equivalent model* reflecting the situation in which all the items in the test have the same units of measurement. These results are congruent with those found by Ximénez (2006), where recovery of weak factor loadings was poor for correctly specified one-factor CFA models in all sample sizes. Thus, adding the associated mean structure to the covariance model favors the recovery of weak factor loadings, even with small sample sizes. Finally, the estimation method also produced a statistically significant effect but its effect size was quite small (η^2^ = 0.001). Congruent with previous research, the results indicate that the recovery of weak factor loadings with the ULS estimation method is slightly better than with the ML method for the smallest sample size (*N* = 100). In addition, in no case did ML outperform ULS in the recovery of weak factor loadings.

In the two-factor model, the largest effects found are also due to the *constraints imposed in the model* (η^2^ = 0.26) and *sample size* (η^2^ = 0.22) main effects. Furthermore, the *N*x*C* and *C*x*CO* interactions also produced a medium effect (η^2^ = 0.15 and 0.12, respectively). Figures 1B,C illustrate the *N*x*C* and *C*x*CO* interactions. As can be seen, adding the associated mean structure improves the recovery of weak factor loadings. The average values of *RMSD* for the CFA model are indicative of a good recovery in all cases except for models with orthogonal factors and with sample sizes of *N* = 100 (see Table 2). Thus, the presence of factor correlation improves the recovery of weak factor loadings in the CFA model. However, this is not the case for the models with the addition of the associated mean structure as the recovery of weak factor loadings is very similar in the models with orthogonal and correlated factors. Concerning the effect of the constraints imposed in the mean structure, and similar to the case with the one-factor model, the best results are for C2, which reflects the situation in which all items in the test have the same units of measurement. The recovery of weak factor loadings for the items having the same units of measurement only in the weak factor (C4) also shows a satisfactory recovery, whereas recovery worsens in the saturated model (C1) and in the model having items with the same units of measurement only in the strong factor (C3) when the sample size is *N* = 100. Overall, the results for the two-factor model show that adding the associated mean structure favors the recovery of weak factor loadings, which is satisfactory even with small sample sizes. In addition, congruent with previous research, the recovery of weak factor loadings in CFA models not including the mean structure worsens if the factors are orthogonal and is especially poor for the smallest sample size. However, the results show that if the model includes the associated mean structure, it is not necessary to define the factors as correlated for the adequate recovery of the weak factor loadings. In this case, the effect of the estimation method is quite small but favors the use of ULS estimation with small sample sizes.

Finally, in the three-factor model, as in the two-factor model, the largest effects are attributable to the *constraints imposed in the model* and *sample size* main effects and to the *N*x*C* and *C*x*CO* interaction effects (η^2^ = 0.11). Figures 1D,E illustrate the *N*x*C* and *C*x*CO* interactions. As can be seen, adding the mean structure to the CFA model favors the recovery of weak factor loadings, particularly for C2 (all items in the test have the same units of measurement) and C5 (the items in the weak factor have the same units of measurement). Furthermore, for small sample sizes (*N* = 100), the recovery is satisfactory only when the CFA model includes the mean structure. Finally, the results show that when the model includes three factors, one being a weak factor, it is important to define the factors as correlated. If factors are defined as orthogonal, adding the associated mean structure improves the recovery of weak factor loadings, especially if all items have the same units of measurement (constraint C2) and if the sample size includes 300 observations or more.

### ARE

**Figure 2:**
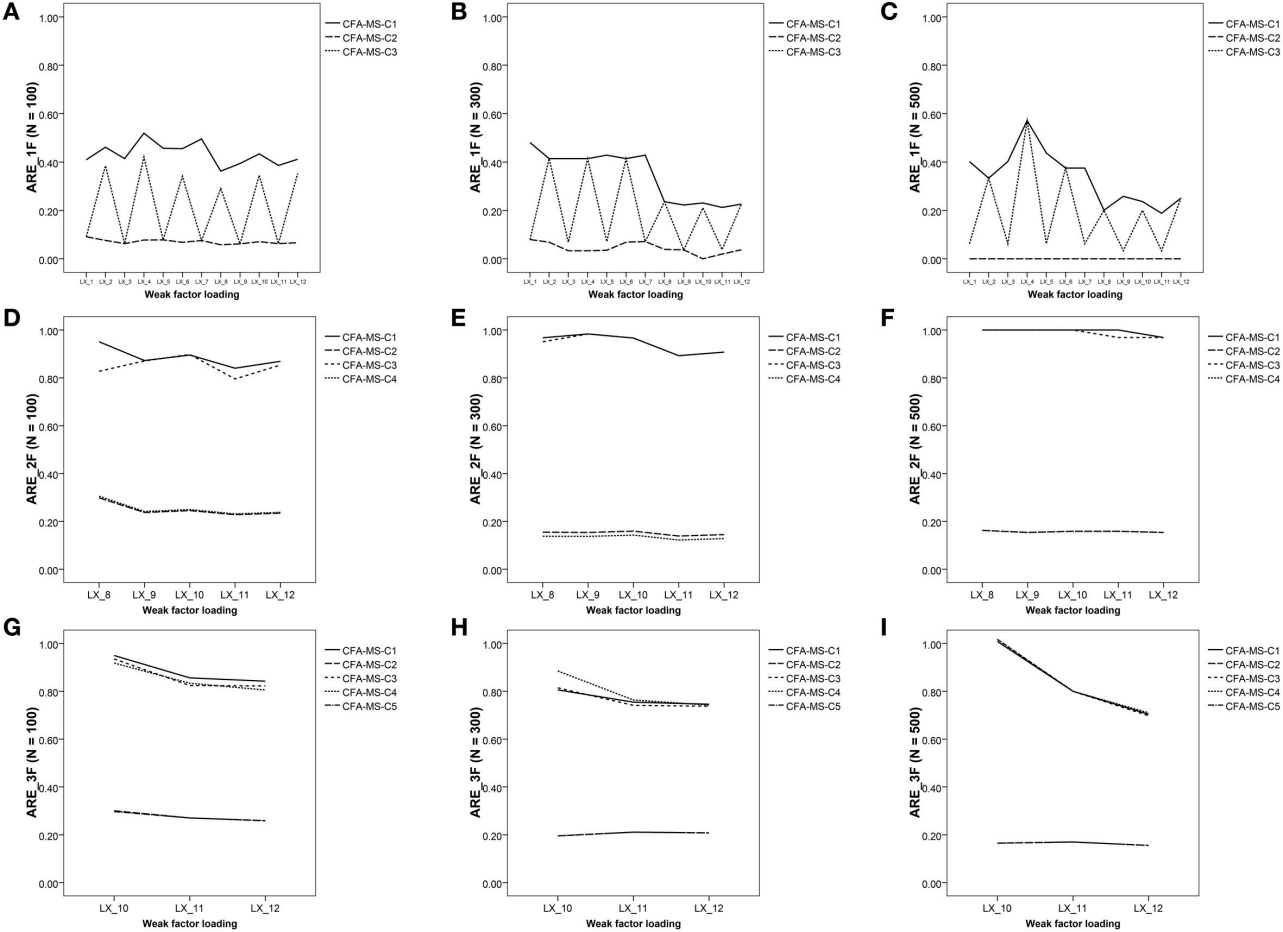
**Graphical representation of *ARE* values in the weak factor loadings of the different model**. **(A)** ARE values in 1F model (*N* = 100); **(B)** ARE values in 1F model (*N* = 300); **(C)** ARE values in 1F model (*N* = 500); **(D)** ARE values in 2F model (*N* = 100); **(E)** ARE values in 2F model (*N* = 300); **(F)** ARE values in 2F model (*N* = 500); **(G)** ARE values in 3F model (*N* = 100); **(H)** ARE values in 3F model (*N* = 300); **(I)** ARE values in 3F model (*N* = 500).

The summary statistics for *ARE* for all main effects and the ANOVA results appear in Tables 2, 4. In the one-factor model, as shown in Table 2, all the *ARE* mean values are less than 1, indicating that adding the associated mean structure to the model reduces the asymptotic variances for the weak factor loadings in all the study conditions. Figure 1F illustrates the *N*x*C* interaction, indicating that the reduction in the asymptotic variance occurs in all the constraints defined for the mean structure regardless of the sample size. In addition, the reduction is substantial for the C2 constraint, which reflects the tau-equivalent model. Figures 2A–C show the mean values of *ARE* for each item in the model. Although all graphs show that there is a reduction of asymptotic variances for the factor loadings in all the study conditions, the best results are for the tau-equivalent model (C2), in which the reduction is substantial. These results are congruent with the study by Yung and Bentler (1999) for one-factor models in a single group, which showed that adding the associated mean structure is especially beneficial for the estimation of factor loadings with smaller true values.

**Table 4:** **ANOVA Results for the *ARE* measure in the Monte Carlo study**.

	**One-factor model**				**Two-factor model**				**Three-factor model**			
	***df***	***F***	***Prob***.	**η^2^**	***df***	***F***	***Prob***.	**η^2^**	***df***	***F***	***Prob***.	**η^2^**
*M*	1	0.820	0.368	0.012	1	34.259	< 0.001	0.407	1	0.556	0.463	0.021
*N*	2	5.242	< 0.001	0.137	2	3.676	0.032	0.128	2	1.928	0.166	0.129
*C*	2	200.972	< 0.001	0.753	3	6113.219	< 0.001	0.992	4	430.383	< 0.001	0.943
*CO*	–	–	–	–	1	103.295	< 0.001	0.674	1	68.886	< 0.001	0.726
*M* ^*^ *N*	2	0.141	0.869	0.004	2	14.839	< 0.001	0.372	2	3.442	< 0.001	0.209
*M* ^*^ *C*	2	0.218	0.805	0.003	3	2.694	0.048	0.051	4	0.703	0.592	0.026
*M* ^*^ *CO*	–	–	–	–	1	0.208	0.650	0.004	1	0.039	0.845	0.001
*N* ^*^ *C*	4	1.273	0.028	0.037	6	5.139	< 0.001	0.667	8	1.342	0.023	0.094
*N* ^*^ *CO*	–	–	–	–	2	23.624	< 0.001	0.486	2	1.279	0.029	0.090
*C* ^*^ *CO*	–	–	–	–	3	5.130	0.002	0.093	4	1.587	0.183	0.058
Error	66	(0.015)			200	(0.006)			130	(0.056)		
Total	79			0.946	224			0.996	159			0.977

Where M is method (ML vs. ULS), N is sample size (100, 300, or 500), C is type of constraint (see Table 1), and CO is correlation between factors (0 or 0.50). Values in parentheses represent mean squared errors.

In the two-factor model, one being strong and the other one weak, all *ARE* values are less than 1, except those of the saturated model (C1) with ML estimation. The largest effects found are due to the constraints imposed in the model (η^2^ = 0.99), factor correlation (η^2^ = 0.67), and estimation method (η^2^ = 0.41) main effects and to the *N*x*C, N*x*CO*, and *M*x*N* interactions, which are graphically represented in Figures 1G–I. As can be seen, the constraints that produce the largest reduction in the asymptotic variances for the weak factor loadings are C2 and C4, whereas the *ARE* values for C1 and C3 are close to 1 (for more details see Figures 2D–F). In this case, the reduction in the asymptotic variance is larger for models with factors defined as orthogonal and small sample sizes (*N* = 100). Additionally, the ULS method produces better results, particularly for the sample size *N* = 100. Thus, adding the mean structure to the two-factor model is more beneficial when using small sample sizes and models with orthogonal factors.

Finally, the results in the three-factor model are very similar to those of the two-factor model (see Figures 1J–L, 2G–I). The largest effects found are due to the constraints imposed in the model (η^2^ = 0.94) and factor correlation (η^2^ = 0.73) main effects, and to the *M*x*N* interaction (η^2^ = 0.21). The reduction in the asymptotic variance is substantial for the C2 and C5 constraints, which imply assuming the same units of measurement in all items or at least in the items of the weak factor. The reduction in the asymptotic variance for the weak factor loadings is larger for the models with the factors defined as orthogonal and for ULS estimation with small sample sizes (*N* = 100).

## Summary and discussion

This article has presented the results of a simulation study aimed at examining the conditions that affect the recovery of weak factor loadings when the CFA model includes the mean structure, compared to analyzing the covariance structure alone. This study contributes to previous research in three ways. First, the impact of modeling the means on the recovery of weak factor loadings had not previously been studied, and this study has specifically addressed this issue. This represents a realistic condition for researchers and practitioners because classical measurement models make assumptions involving latent means and covariance structures, and many CFA models incorporating the mean structure are used in practice. Second, previous research has found that adding the mean structure to the covariance model reduces the asymptotic variances for factor loadings (Yung and Bentler, 1999) but has not provided evidence concerning the different conditions that favor this reduction, and this study considers several conditions to explore the cases in which it could be better to model the means. And third, the study provides practical implications for the use of CFA with factorial structures that include weak factor loadings and incorporating the mean structure, which can be useful for applied researchers and practitioners.

Overall, the results of the study indicate that adding the mean structure to the covariance model affects the recovery of weak factor loadings and that certain conditions are important for the design of the study. First, the recovery of weak factor loadings improves when adding the associated mean structure to the CFA model, particularly if the constraints imposed on the mean structure imply that all the items have the same units of measurement. Second, the definition of the sample size is important when the model includes weak factor loadings (the models with the covariance structure alone require a minimum of 300 or 500 observations for adequate recovery); in contrast, recovery of weak factor loadings is satisfactory when the mean structure is added to the covariance model regardless of the sample size. Third, the recovery of weak factor loadings in CFA models not including the mean structure worsens if the factors are orthogonal, and is especially poor for small sample sizes. However, if the model includes the associated mean structure, it is not necessary to define factors as correlated for adequate recovery. Fourth, ULS performs slightly better than ML and recovers the weak factor loadings in some instances in which ML fails. Fifth, non-convergent solutions and Heywood cases increase when the factors are orthogonal, the sample size is small, and the model does not include the mean structure; in addition, there were more convergent solutions with the ULS method. Finally, the results indicate that the reduction in the asymptotic variance for the factor loadings occurs for all the constraints imposed in the mean structure, and that it is substantial when the constraints imply that all the items have the same units of measurement. In addition, the reduction is larger for models with factors defined as orthogonal and small sample sizes, particularly when using ULS estimation. These results are consistent with those found in the studies by Ximénez (2006) and Yung and Bentler (1999) but provide further understanding of other conditions of the design of the study that affect the estimation of the parameters when adding the mean structure to the covariance model, which had not been studied before.

At one level, the results of this study provide insights about the recovery of weak factor loadings in CFA when the model includes the mean structure. At another level, some results have implications for the practical use of CFA with factorial structures that include weak factor loadings and incorporating the mean structure, a situation which is present to some degree in practice. These issues have to do with aspects of the design of a study:

1. This study has demonstrated how important it is to incorporate the mean structure in the model for the adequate recovery of weak factor loadings, particularly if all the items in the test have the same units of measurement. Of course, theoretical aspects must be considered when deciding whether variable means should be modeled or not. However, researchers and practitioners should be aware that for models including weak factor loadings, modeling the associated mean structure in conjunction with the covariance structure should be seriously considered. Therefore, it is desirable for researchers and practitioners to design their questionnaires containing items with the same units of measurement and to analyze the dimensionality of the test with CFA models that include the associated mean structure. This piece of advice is important because in practice it is frequent to find questionnaires using items with different units of measurement, and researchers and practitioners habitually analyze their models using only the covariance structures.
2. The present study has also demonstrated that the constraints imposed on the classical measurement *tau-equivalent* model derived from classical test theory (Jöreskog, 1971) improve the recovery of weak factor loadings. Moreover, similar results are expected for the *parallel* model, as it also follows the Yung and Bentler (1999) framework. Applied researchers may have substantive reasons to estimate models that include weak factors, as in the hierarchical model from Ackerman (1996). The results of this article show that the estimation of such factors will be improved if data conform to the *parallel* or *tau-equivalent* models. Thus, researchers can proceed by testing the goodness of fit for *parallel* or *tau-equivalent* models following the procedures by Millsap and Everson (1991); if one of these models provides satisfactory fit, it will render more precise estimates of the weak factor than the traditional factorial model with unconstrained mean structure.
3. Although, previous research suggests that sample size must be larger than typically recommended when the model includes weak factor loadings, this study has revealed that if the analysis of the mean structure is included, it is not necessary to work with large sample sizes. The factor analysis literature contains a variety of recommendations regarding the sample size to be used for conducting a factor analysis. For the most part, these recommendations are presented as a suggested minimum sample size depending on the number of factors in the model, the number of variables per factor, and the level of communality. Mundfrom et al. (2005) offered a table with specific recommendations of minimum necessary sample sizes for models from one to six factors and different levels of communalities. For example, a two-factor model with low levels of communality (from 0.20 to 0.40) needs a minimum of 150 observations if there are five variables per factor, of 270 observations if there are four variables per factor, and of 900 observations if there are three. Thus, a small number of variables per factor requires a larger minimum sample size than does a large number of variables. The results of the present study suggest that, for CFA models with mean structure, 100 observations is a sufficient sample size for the adequate factor recovery, even with a ratio of three variables per factor and small factor loadings. However, given that the scope of the results is limited to the particular conditions considered in the simulation study, further study should be devoted to determine whether the general rules of thumb regarding sample size can be followed when the analysis includes the mean structure. In addition, given that the minimum sample size considered here was 100 observations, further study is needed to determine whether these results hold with smaller sample sizes (e.g., those considered in the study by De Winter et al., 2009).
4. Whereas previous research on the recovery of weak factor loadings has suggested that factors should be defined as correlated for adequate recovery, this is not necessary when the model includes the associated mean structure in conjunction with the covariance structure. Therefore, researchers and practitioners can define their factorial models with orthogonal factors and analyze the dimensionality problem by means of CFA models with mean structure.
5. Finally, this study has also demonstrated that when the data come from a population structure in which all factors are not equally strong, ML fails to recover the weak factor in some instances in which ULS succeeds. Therefore, researchers should favor the use of ULS estimation, or should at least compare the ML and ULS solutions. Previous research had already suggested the superiority of ULS over ML on the recovery of weak factor loadings. However, this represents an important piece of advice for applied researchers who, in many cases, erroneously believe that under normality, ML is the only available method for estimating a CFA model.

Overall, the present study has shown that working with factorial structures including small factor loadings is not necessarily a problem. Such models exist in practice and are commonly found in psychological research, and they can be reproduced with similar properties as the models with larger factor loadings.

### Limitations and directions for future study

As is the case with any Monte Carlo simulation study, the results found in this research will hold only in conditions similar to those considered here. Thus, future research should continue examining these effects under different study conditions. For instance, first, further study could be directed to examining whether the magnitudes of the effects found here hold under conditions of model error. This condition was not examined here because it exceeded the scope of the study and because previous research has found that recovery of weak factor loadings is unaffected by model error (Ximénez, 2009). However, this is a topic of interest because models are always wrong to some degree. Second, given that the particular manner in which the constraints on the mean structure were formulated probably had an impact on the results, other studies should continue examining the effects found here by defining the constraints on the mean structure in other ways. Finally, another potential line of research could be to examine the recovery of weak factor loadings in the context of other CFA models. For instance, in multiple-group designs, which involve a more complex situation than CFA, and in bifactor measurement models, which potentially provide a solid foundation for conceptualizing psychological constructs, constructing measures, and evaluating a measure's psychometric properties (Reise, 2012), and where it is frequent to work with small factor loadings.

### Conflict of interest statement

The author declares that the research was conducted in the absence of any commercial or financial relationships that could be construed as a potential conflict of interest.
